# On the suitability of additively manufactured gyroid structures and their potential use as intervertebral disk replacement - a feasibility study

**DOI:** 10.3389/fbioe.2024.1432587

**Published:** 2024-07-22

**Authors:** Valentin Gross, Sergej Zankovic, Bernd Rolauffs, Dirk Velten, Hagen Schmal, Michael Seidenstuecker

**Affiliations:** ^1^ G.E.R.N. Tissue Replacement, Regeneration and Neogenesis, Department of Orthopedics and Trauma Surgery, Medical Center-Albert-Ludwigs-University of Freiburg, Faculty of Medicine, Albert-Ludwigs-University of Freiburg, Freiburg, Germany; ^2^ Institute for Applied Biomechanics, Offenburg University, Offenburg, Germany; ^3^ Department of Orthopedics and Trauma Surgery, Medical Center-Albert-Ludwigs-University of Freiburg, Faculty of Medicine, Albert-Ludwigs-University of Freiburg, Freiburg, Germany

**Keywords:** additive manufacturing, 3D-printing, fused deposition modeling FDM, gyroid, intervertebral disk, mechanical properties

## Abstract

**Introduction:**

Intervertebral disk degeneration is a growing problem in our society. The degeneration of the intervertebral disk leads to back pain and in some cases to a herniated disk. Advanced disk degeneration can be treated surgically with either a vertebral body fusion or a disk prosthesis. Vertebral body fusion is currently considered the gold standard of surgical therapy and is clearly superior to disk prosthesis based on the number of cases. The aim of this work was the 3D printing of Gyroid structures and the determination of their mechanical properties in a biomechanical feasibility study for possible use as an intervertebral disc prosthesis.

**Material and methods:**

Creo Parametric 6.0.6.0 was used to create models with various Gyroid properties. These were printed with the Original Prusa i3 MK3s+. Different flexible filaments (TPU FlexHard and TPU FlexMed, extrudr, Lauterach, Austria) were used to investigate the effects of the filament on the printing results and mechanical properties of the models. Characterization was carried out by means of microscopy and tension/compression testing on the universal testing machine.

**Results:**

The 3D prints with the FlexHard and FlexMid filament went without any problems. No printing errors were detected in the microscopy. The mechanical confined compression test resulted in force-deformation curves of the individual printed models. This showed that changing the Gyroid properties (increasing the wall thickness or density of the Gyroid) leads to changes in the force-deformation curves and thus to the mechanical properties.

**Conlcusion:**

The flexible filaments used in this work showed good print quality after the printing parameters were adjusted. The mechanical properties of the discs were also promising. The parameters Gyroid volume, wall thickness of the Gyroid and the outer wall played a decisive role for both FlexMed and FlexHard. All in all, the Gyroid structured discs (Ø 50 mm) made of TPU represent a promising approach with regard to intervertebral disc replacement. We would like to continue to pursue this approach in the future.

## 1 Introduction

Disk degeneration is an increasing problem in today’s society. Disk degeneration is usually the starting point for a later herniated disk. Physical strain or overload and the normal ageing process of the body can lead to disk degeneration, as well as illness and injury. The fibrous ring becomes fissures over time and develops small tears. Fluid can leak out of the gelatinous core through these cracks. This makes it increasingly difficult to absorb shocks and can eventually lead to a bulging or herniated disk (Abudouaini et al., 2023). If conservative treatment is no longer possible, the damaged disk must be surgically removed and treated using one of the following two methods. Spinal fusion (spondylodesis, vertebral body fusion) is now considered the gold standard in the surgical treatment of cervical and lumbar disk degeneration (Abi-Hanna et al., 2018; Abudouaini et al., 2023; Goedmakers et al., 2023). However, this procedure also involves certain risks, such as limited mobility of the spine and increased stress on the neighboring vertebral segments. This in turn can lead to accelerated degeneration of the adjacent disks (adjacent segment degeneration (ASD)) and cause pain in the affected region as well as compression of the surrounding nerves (Gornet et al., 2017; Cui et al., 2018; Li et al., 2022; Abudouaini et al., 2023; Goedmakers et al., 2023). The use of disk prostheses for disk degeneration is a slowly emerging alternative to cervical and lumbar fusion. The disk prosthesis makes it possible to maintain the natural ROM (range of motion) of the disk and reduce the risk of ASD (Feng et al., 2017; Pham et al., 2018; Patwardhan and Havey, 2020; Shen et al., 2021). According to the Federal Statistical Office, spondylodeses and implanted disk prostheses operated on in Germany in the years 2020–2022 (Bundesamt, 2024). It can be seen here that the number of spondylodeses operated on increased by more than 2000 operations from 2020 (67,380) to 2021 (69,728). The number of implanted disk prostheses is significantly lower than that of spondylodeses. In 2020, 4,415 disk prostheses were implanted in Germany, in the following year 2021 there were 4,205 and in the next year 2022 only 3,978.

When considering the Young’s modulus of the human intervertebral disc, a distinction must be made between the annulus fibrosus with values in the range of 4–8 MPa and the nucleus pulposus, whose Young’s modulus is typically in the range of 0.1–0.5 MPa (Cloyd et al., 2007). In contrast, the Young´s moduli of the disc replacement are significantly higher, especially for the metallic components such as titanium (typically 100–200 GPa) and the polymer components (PE, PU) in the range 0.5–3 GPa) (Warburton et al., 2020). Additive manufacturing is becoming increasingly popular in the medical field, and intervertebral disk cages can already be manufactured individually for each patient, with a very precise fit (Serra et al., 2016). The availability of basic materials for additive manufacturing, such as flexible thermoplastic polyurethane (TPU) filaments, is also increasing (Xiao and Gao, 2017). In this context, we wanted to investigate whether additively manufactured Gyroid constructs made of flexible TPU filament are potentially suitable for use as intervertebral disk replacements.

## 2 Materials and methods

### 2.1 Additive manufacturing

Creo Parametric 6.0.6.0 (PTC, Boston, Massachusetts, United States) was used to create the 3D models. To simplify the process and the experiments, the Gyroid structures were created as cylinders with a diameter of 50 mm and a height of 10 mm. The Gyroid was varied from 10 mm³ for the coarsest structure to 4 mm³ for the finest structure. The wall thickness of the Gyroid was also varied from 0.5 to 1.0 mm. The outer wall of the disk has a wall thickness of 0.4–0.8 mm. A summary of the individual parameters is shown in Table 1 below. These 36 different variations were used for both FlexHard and FlexMed.

**TABLE 1:** Gyroid Dimensions for additive manufacturing.

Sample	Gyroid dimensions		
	Volume [mm³]	Wall thickness gyroid [mm]	Wall thickness [mm]
FH01	10	0.5	0.4
FH02	10	0.75	0.4
FH03	10	1.0	0.4
FH04	8	0.5	0.4
FH05	8	0.75	0.4
FH06	8	1.0	0.4
FH07	6	0.5	0.4
FH08	6	0.75	0.4
FH09	6	1.0	0.4
FH10	4	0.5	0.4
FH11	4	0.75	0.4
FH12	4	1.0	0.4
FH13	10	0.5	0.6
FH14	10	0.75	0.6
FH15	10	1.0	0.6
FH16	8	0.5	0.6
FH17	8	0.75	0.6
FH18	8	1.0	0.6
FH19	6	0.5	0.6
FH20	6	0.75	0.6
FH21	6	1.0	0.6
FH22	4	0.5	0.6
FH23	4	0.75	0.6
FH24	4	1.0	0.6
FH25	10	0.5	0.8
FH26	10	0.75	0.8
FH27	10	1.0	0.8
FH28	8	0.5	0.8
FH29	8	0.75	0.8
FH30	8	1.0	0.8
FH31	6	0.5	0.8
FH32	6	0.75	0.8
FH33	6	1.0	0.8
FH34	4	0.5	0.8
FH35	4	0.75	0.8
FH36	4	1.0	0.8

*FH … FlexHard.

**FIGURE 1:**
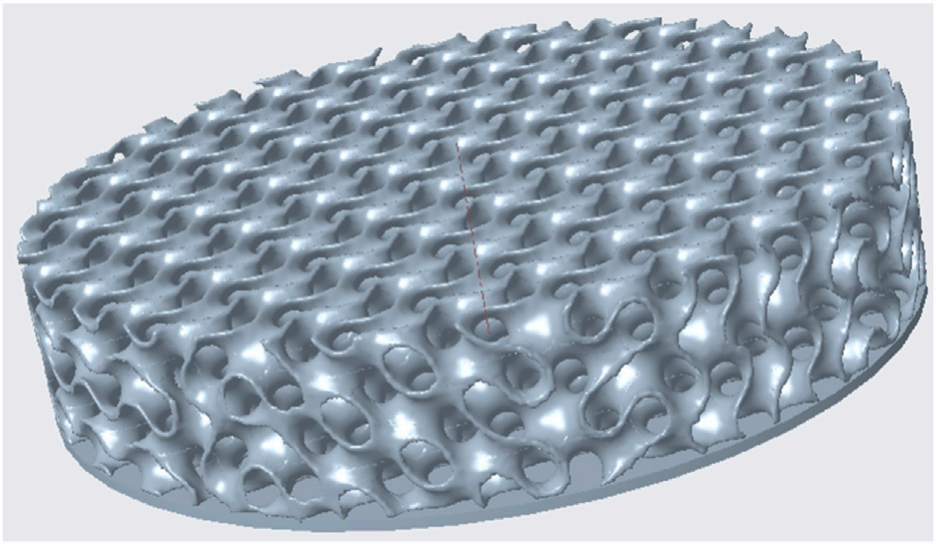
Example illustration of the Gyroid structure created with Creo Parametric, but without the outer wall for better visualization.

**FIGURE 2:**
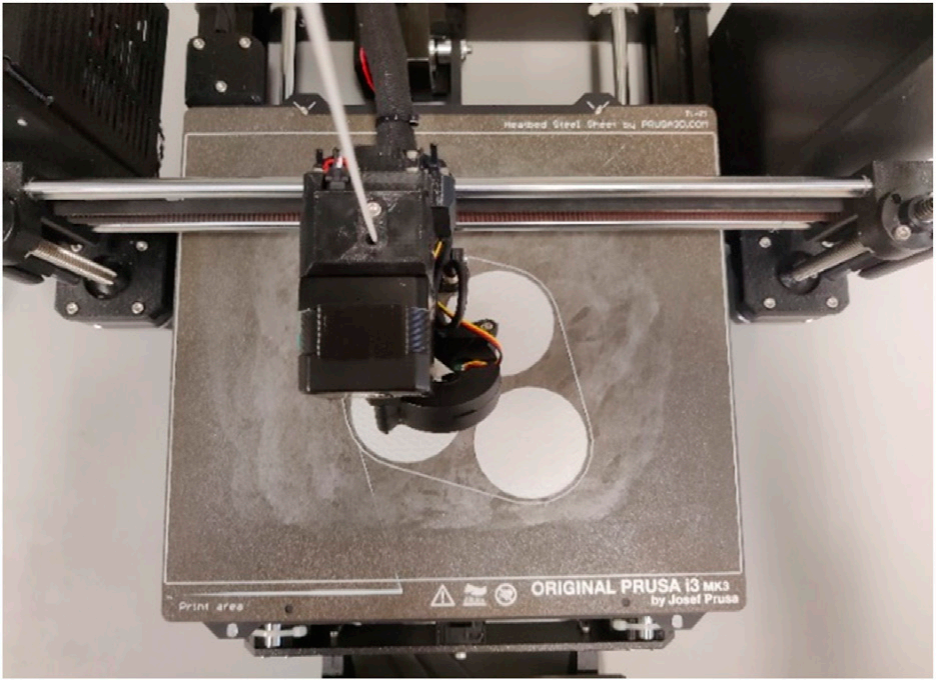
Top view of the printing plate with a visible adhesive layer to improve adhesion to the printing plate around the printed parts.

All models (see Figure 1) created were exported as STL files and loaded into PrusaSlicer 2.7.4 (PRUSA Research, Praque, Czech Republic) and prepared for 3D printing on the Prusa i3 MK3S+ (PRUSA Research). It is important to change the filament in the slicer accordingly. The FlexHard filament has a Young´s modulus of 40 MPa. The general printing parameters remain unchanged, only the nozzle and bed temperatures change. The FlexHard and later also FlexMed filament had been printed at a nozzle temperature of 240°C and a bed temperature of 50°C. Once all parameters have been set in the PruseSlicer, the model is sliced. This calculated how long the print will take and how much material will be used during printing. The file created by slicing was exported as a G-code file and transferred to the Pruse i3 MKS+. The TPU filaments FlexHard and FlexMed were purchased by extrudr.com (FD3D GmbH, Lauterach, Austria). FlexHard in white and FlexMed in blue were purchased for color differentiation. It was printed on the textured spring steel plate with a large area of the printing bed coated with a glue stick (Kores, Vienna, Austria) to ensure optimum adhesion of the TPU filament (see Figure 2).

### 2.2 Characterization

#### 2.2.1 Microscopy

The 3D-printed disks were examined using an Olympus stereomicroscope SZ61 (Olympus Inc., Tokyo, Japan) equipped with Olympus SC30 camera and × 6.7 magnification. For this purpose, special specimen were printed that were only half the height of the disks produced for the mechanical tests. The 3D printing of the slices was interrupted at 50% to better visualize the internal structure. The slices were not cut because the cutting process itself could cause errors. The thickness and spacing of the Gyroid were measured at least five different points on each cylinder and compared with the target values from the STL.

#### 2.2.2 Mechanical properties

In accordance with DIN EN ISO 527–1, a tensile test was carried out with 3D printed specimens 5 A. The aim was to investigate the difference between FlexMed and FlexHard. The tensile tests were carried out on the Z005 universal testing machine (Zwick-Roell GmbH and Co. KG, Ulm, Germany) with at least five specimens each. In order to test the mechanical properties of the 3D-printed parts, an unconfined compression test was carried out with at least three specimen of each setting. The body was compressed to 50% of its total height and the force required was recorded in the form of a force-deformation curve. The unconfined compression test (in accordance with DIN EN ISO 527–1 for plastics) was performed using the Zmart. Pro universal testing machine (Zwick-Roell GmbH & Co. KG, Ulm, Germany). The initial position was first set, which later served as a reference point. A preload of 1 N was then set in the testing machine settings, at which the measurement should begin. The body to be measured was positioned between the two measuring plates of the testing machine. After checking that the force has been set to zero, the measurement has been started. As soon as the upper plate of the testing machine hits the part and the pre-force of 1 N is exceeded, the software of the testing machine started to record the force-deformation curve. The crosshead speed was set to 5 mm/min. After reaching a deformation of 50%, the measurement was automatically stopped and the two plates moved back to the previously defined starting position. The measurement was also stopped when the specified maximum force of 20 kN was reached. The sampling rate was 1,000 Hz, so an average of 35,000 points were recorded for all force/displacement curves. For subsequent analysis, the results of three samples each were averaged and plotted as force-displacement curves. Exact specimen thickness was measured (at five different points on the specimen) before and after the test using a digital caliper. Sample thickness was also re-determined 24 h after the test. According to Wang and Campbell, (2009), human vertebral endplates can withstand maximum loads of approximately 4,000–6,000 N. In fact, Rohlmann et al. (2001) show that up to 200% of a person’s own body weight can act on the intervertebral disks during the activities of daily living. During exercise, this can be up to 250% of body weight. For a man weighing 85 kg, this corresponds to an axial load of approximately 2100 N on the intervertebral disks. In order to cover these forces and to be able to withstand possible force peaks, e.g., in the event of a fall, a target range was set for the 3D-printed disks between 4,000 and 7,500 N as the load limit for the mechanical examinations (Wang and Campbell, 2009). Exclusion criteria were a maximum force greater than 7,500 N or a compressive strength greater than 3.81 MPa (based on 50 mm diameter). The maximum force was chosen to be slightly higher than in Wang et al. (Wang and Campbell, 2009) to compensate for the larger area of the disc used compared to the actual area of an intervertebral disc. Thus, the investigations were in a similar measuring range (up to 3.5 MPa) as the measurements of Wilke et al. (Wilke et al., 1999) in an intact L4/5 disc of a 45-year-old man.

### 2.3 Statistics

All results are expressed as means ± standard deviations. The measured values were analyzed using ANOVA (Tukey) with a significance level of *p* < 0.05, after testing for normal distribution using the Shapiro-Wilk test. Origin 2023 Professional SR1 (OriginLab, Northampton, MA, United States) was used for all statistical analyses.

## 3 Results

### 3.1 Sample dimensions

**FIGURE 3:**
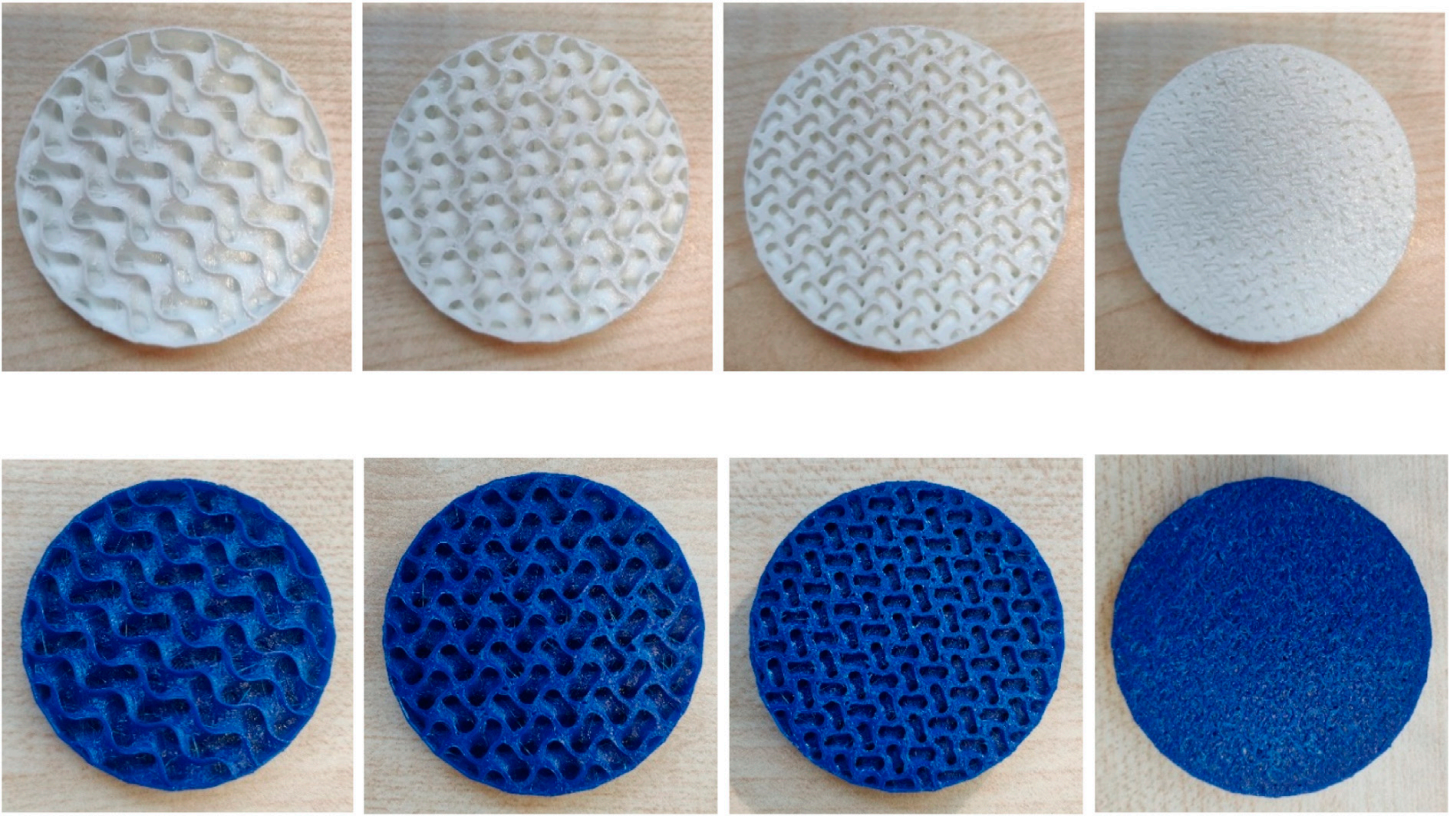
Exemplary images of samples made of FlexHard (white) and FlexMed (blue) with variation of the Gyroid volume from 10 mm³ on the left to 4 mm³ on the right, the second and third image in each row represents 8 and 6 mm³ respectively, sample diameter 50 mm.

Figure 3 below shows some examples of Gyroid structures from FlexHard and FlexMed.

**FIGURE 4:**
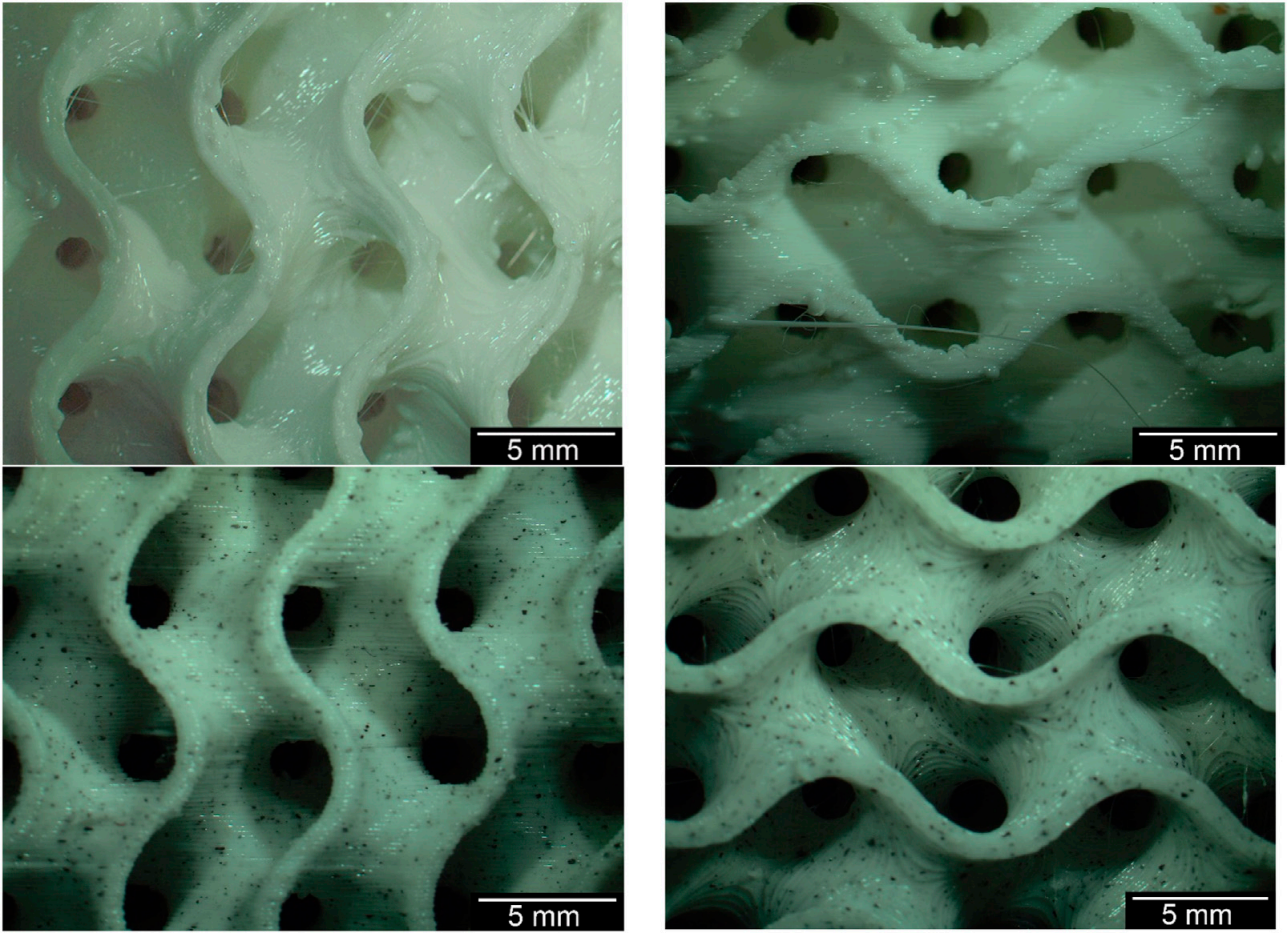
Gyroid structures before (top) and after (bottom) the optimization of the printing temperature including the temperature change of the printing bed.

Without optimizing the printing temperature from 215°C to 240°C and reducing the print bed temperature from 60°C to 50°C, the microscopic view showed various printing defects such as threads or drops on the surface. Some examples are shown in Figure 4 below. These defects could be eliminated by changing the printing parameters and using a filament dryer (Crealty, Shenzhen, China) at 55°C.

**FIGURE 5:**
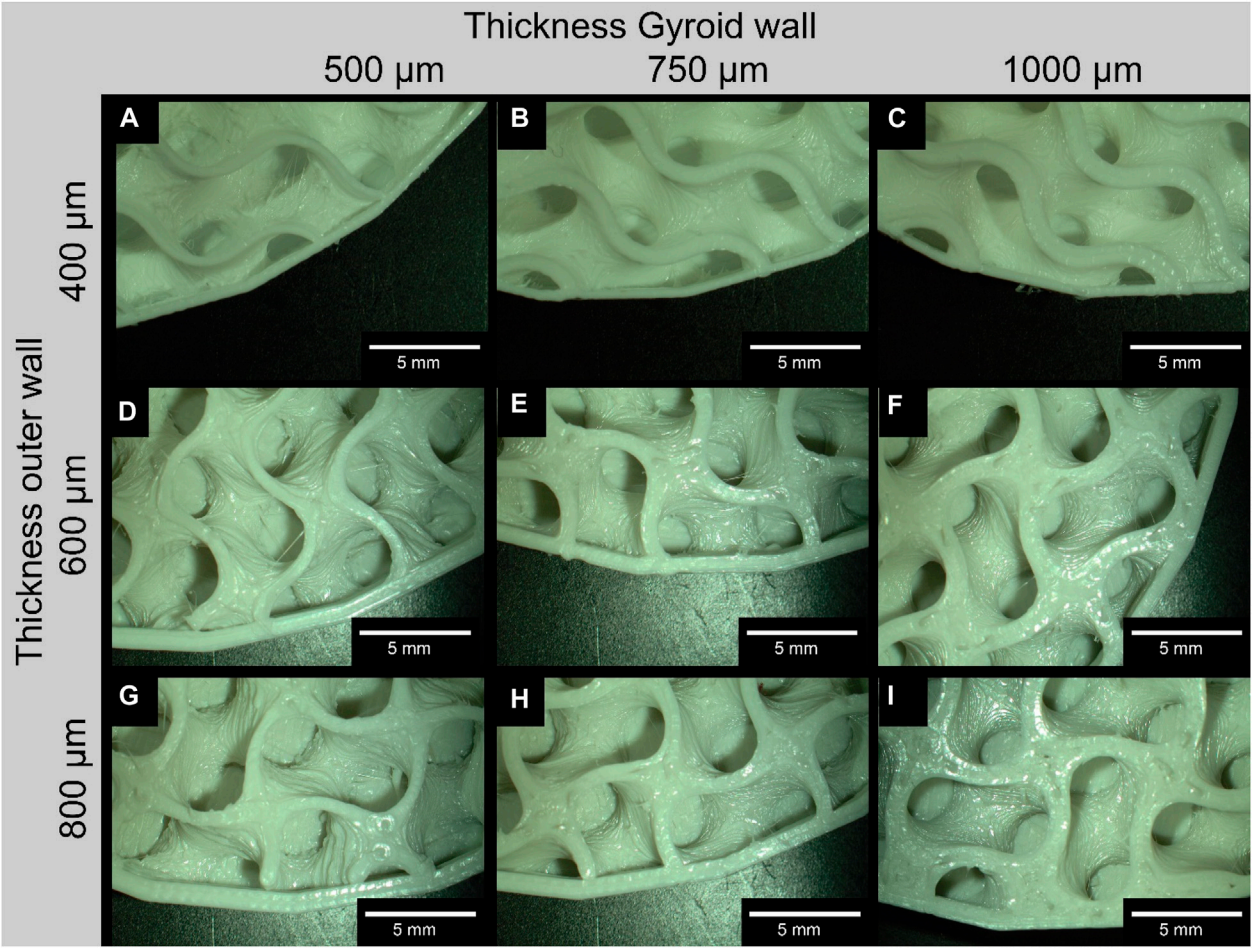
Exemplary comparison of the different wall thicknesses of the Gyroid on the outside and inside; For the images of the disks, the 3D printing was interrupted at 30% to make it easier to measure the wall thicknesses of the Gyroid, otherwise measurement errors could occur due to the curvature (exemplary for a Gyroid volume of 6 mm^3^); **(A, D, G)** represent a gyroid wall thickness of 500 μm; **(B, E, H)** of 750 μm; and **(C, F, I)** of 1000 μm; **(A–C)** represent a thickness of the outer wall of 400 μm; **(D–F)** of 600 μm; **(G–I)** of 800 μm.

An example comparison of the different Gyroid and outer wall thicknesses for a 6 mm³ Gyroid volume is shown in Figure 5. The 3D printing of the slices was interrupted at 50% to better visualize the internal structure. The slices were not cut because the cutting process itself could be a source of error. The comparison of the wall thickness specifications for the Gyroid and outer wall with the measured wall thickness values is summarized in Table 2. Again, we have examined the 6 mm³ Gyroid volume as an example.

**TABLE 2:** Overview of Gyroid and outer wall thicknesses comparison of target and measured values exemplary for a Gyroid volume of 6 mm³ 3D-printed with FlexHard.

Specimen	Gyroid wall (target) [mm]	Gyroid wall (measured) [mm]	Outer wall (target) [mm]	Outer wall (measured) [mm]
FH04	0.5	0.50 ± 0.04	0.4	0.40 ± 0.02
FH05	0,75	0.75 ± 0.05	0.4	0.41 ± 0.04
FH06	1.0	0.99 ± 0.04	0.4	0.40 ± 0.04
FH16	0,5	0.49 ± 0.06	0.6	0.62 ± 0.07
FH17	0.75	0.75 ± 0.05	0.6	0.61 ± 0.03
FH18	1.0	0.98 ± 0.07	0.6	0.61 ± 0.03
FH28	0.5	0.52 ± 0.06	0.8	0.82 ± 0.06
FH29	0.75	0.75 ± 0.04	0.8	0.81 ± 0.06
FH30	1.0	1.00 ± 0.04	0.8	0.79 ± 0.05

### 3.2 Mechanical properties

#### 3.2.1 Tensile tests

The tensile tests were carried out in accordance with ISO EN 527 with 5 A specimens. The measurements were repeated at least 5 times. The FlexMed showed a tensile modulus of 30.2 ± 9.2 MPa while the FlexHard had a higher tensile modulus of 124.1 ± 6.6 MPa. The difference was significant at *p* < 0.05. The following Table 3 summarizes all measured parameters from the tensile test, the other parameters also differ significantly between FlexMed vs. FlexHard.

**TABLE 3:** Summary of the results of the tensile test according to DIN EN ISO 527–1.

FlexMed	Et [MPa]	εY [%]	σY [MPa]	εtB [%]	σB [MPa]
Mean	90.2	374.6	36.6	335.4	36.1
Min	74.8	362.9	34.9	323.9	34.3
Max	101.2	385.7	38.3	350.6	37.5
SD	9.2	7.5	1.3	9.0	1.3

FlexMed	Et [MPa]	εY [%]	σY [MPa]	εtB [%]	σB [MPa]
Mean	124.1	378.7	41.0	319.6	40.6
Min	117.5	358.4	37.9	296.6	37.4
Max	135.0	389.5	44.7	336.0	44.4
SD	6.6	12.0	2.9	15.5	3.0

#### 3.2.2 FlexHard

**FIGURE 6:**
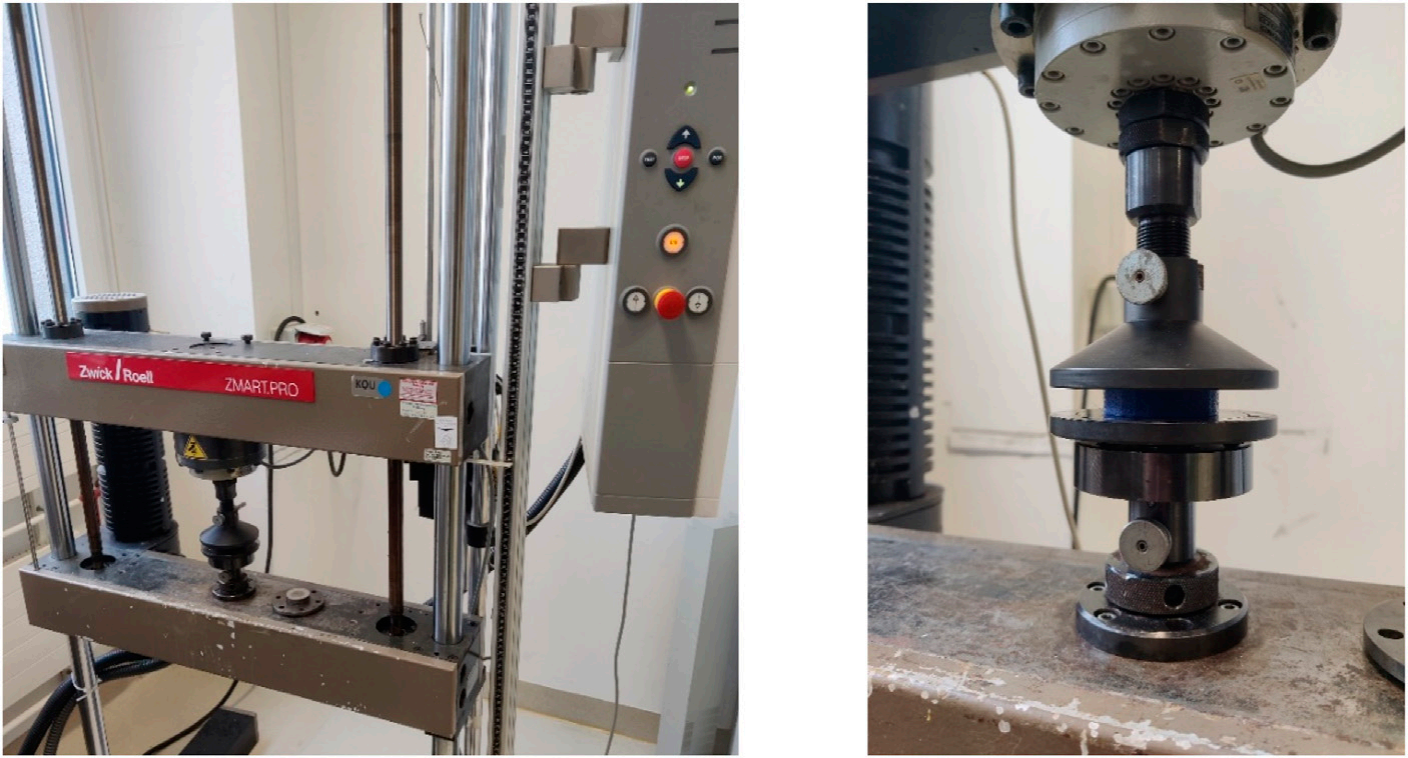
Set-up of the ZwickRoell testing machine (on the left) and execution of the test using FlexMed as an example (on the right).

**FIGURE 7:**
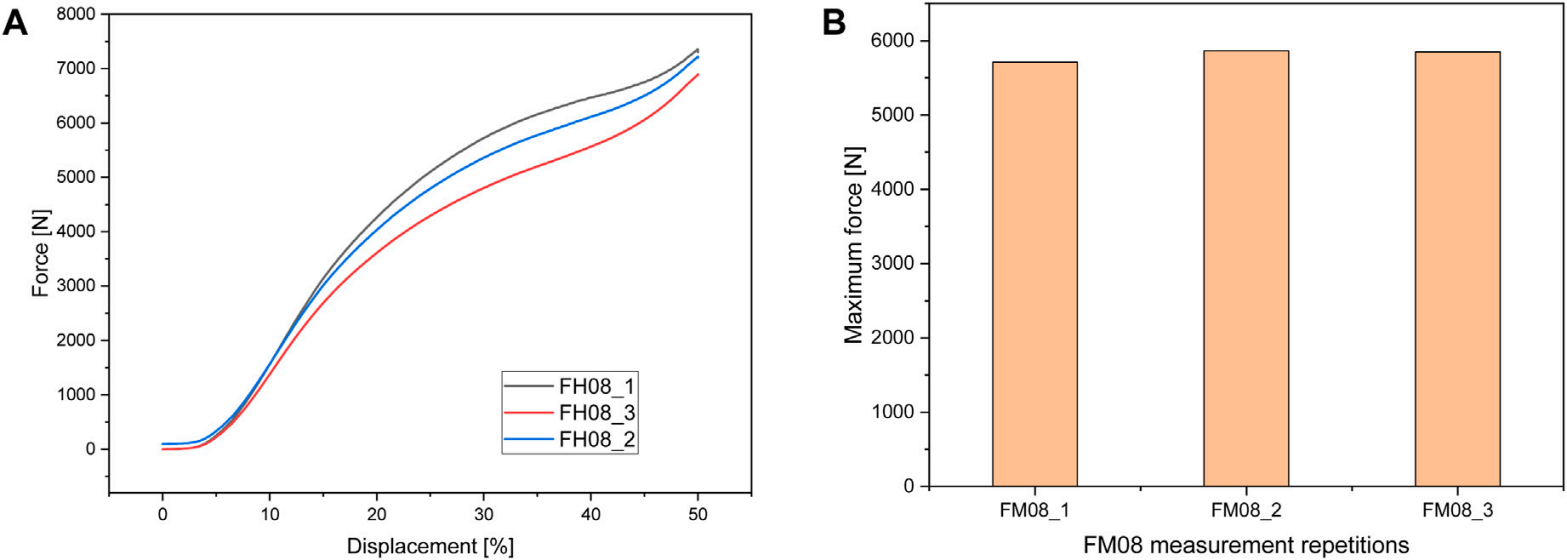
Repeatability of Measurements Illustrated by **(A)** Force/Displacement Curves for FH08 and **(B)** Maximum Force measured for FM08.

**FIGURE 8:**
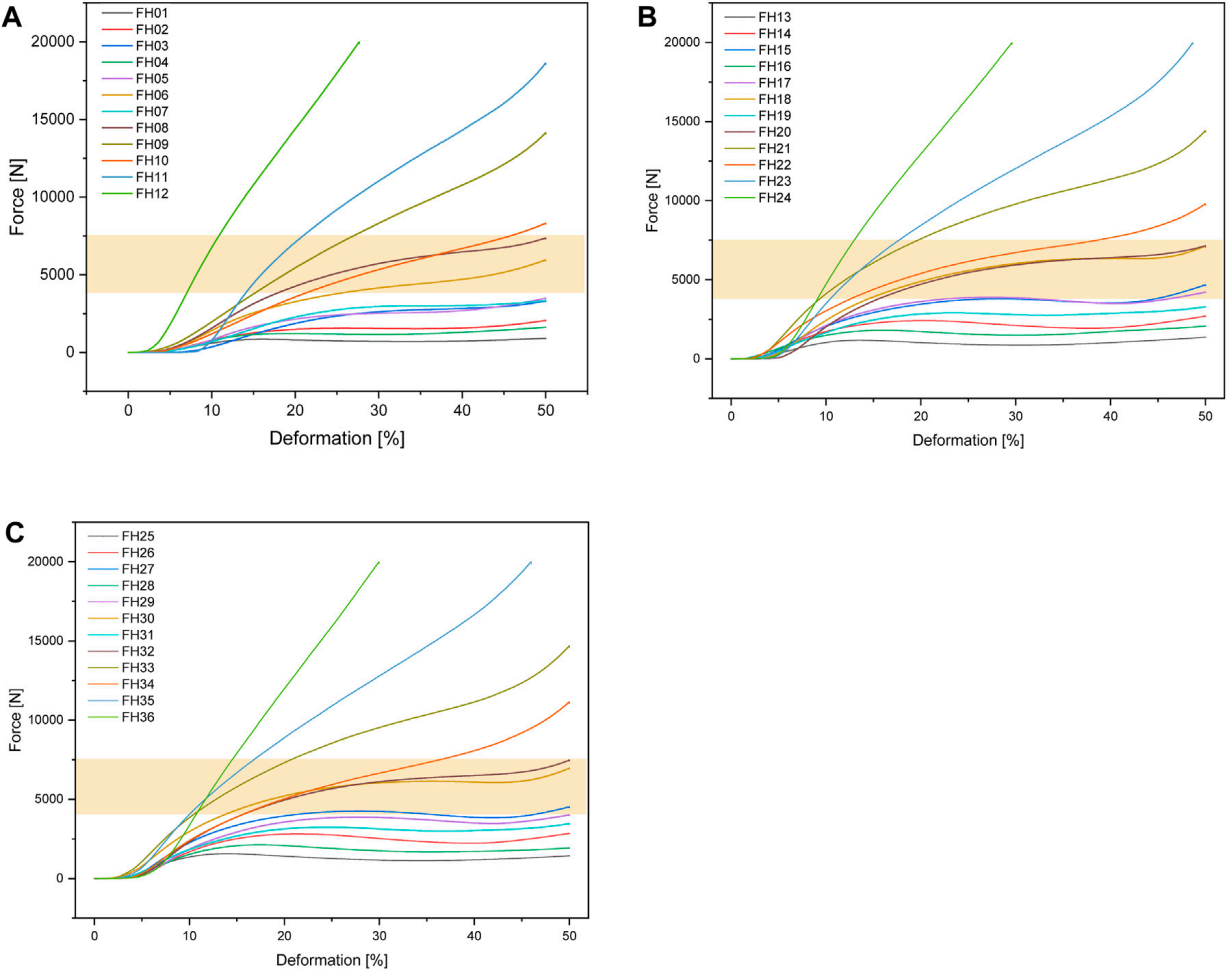
Force-deformation curves for the additively manufactured Gyroid structures out of FlexHard with **(A)** 0.4 mm; **(B)** 0.6 mm and **(C)** 0.8 mm outer wall thickness; orange area: target range for our disks between 4,000 and 7500 N; Approximately 35,000 measurement points for three samples each were averaged for the force/displacement curves.

Figure 6 shows the test setup for the unconfined compression tests. Figure 7 below shows an example of the repeatability of the measurements for samples FH08 and FM08. Figure 7A shows the force/displacement curves of the three measurements and Figure 7B shows the maximum force achieved for FM08 as a bar chart. The overview in Figure 8 shows the results of the mechanical tests. To keep the results clear, we have divided the figure into three diagrams: Figure 8A includes all samples with an outer wall thickness of 0.4 mm, Figure 8B with 0.6 mm and Figure 8C with 0.8 mm. It is clear that a whole series of compositions of the Gyroid structures fall below or exceed the limit between 4,000 and 7,500 N defined by us. For the 0.4 mm wall thickness specimens, only two specimens were within the specified limit: FH06 at 5,909 ± 164 N and FH08 at 7,304 ± 84 N. For the 0.6 mm wall thickness specimens, compression to 50% of the maximum height resulted in maximum forces of 4,632 ± 150 N for FH15, 4,184 ± 103 N for FH17, 7,068 ± 151 N for FH18 and 7,090 ± 175 N for FH20. At 0.8 mm wall thickness, three specimens were within the limit: FH27 at 4,480 ± 80 N, FH30 at 6,910 ± 148 N and FH32 at 7,421 ± 356 N. One specimen, FH29, was just out of specification at 3,983 ± 25 N. All other samples were significantly below or above the limit. Table 4 provides a summary of the FlexHard and FlexMed samples within the limits, including the maximum force achieved.

**TABLE 4:** Overview of Flex specimens within the specified limit with maximum force and compressive strength (N = 3).

FlexHard								
0.4 mm			0.6 mm			0.8 mm		
Sample	Fmax [N]	σ_D_ [MPa]	Sample	Fmax [N]	σ_D_ [MPa]	Sample	Fmax [N]	σ_D_ [MPa]
FH06	5,909 ± 164	3.01 ± 0.08	FH15	4,632 ± 150	2.36 ± 0.08	FH27	4,480 ± 80	2.28 ± 0.04
FH08	7,304 ± 84	3.72 ± 0.04	FH17	4,184 ± 103	2.13 ± 0.04	FH30	6,910 ± 148	3.52 ± 0.07
			FH18	7,068 ± 151	3.60 ± 0.09	FH32	7,421 ± 356	3.78 ± 0.18
			FH20	7,090 ± 175	3.61 ± 0.09			
FlexMed								
FM06	4,475 ± 28	2.28 ± 0.02	FM18	5,699 ± 113	2.90 ± 0.06	FM30	5,504 ± 49	2.80 ± 0.03
FM08	5,864 ± 149	2.99 ± 0.07	FM20	6,068 ± 157	3.09 ± 0.08	FM32	6,338 ± 122	3.23 ± 0.06
FM10	7,024 ± 67	3.58 ± 0.03						

#### 3.2.3 FlexMed

For the Gyroid structures made with FlexMed using additive manufacturing, the same specimens as additive manufactured with FlexHard were within the specified limits. With a wall thickness of 0.4 mm, the samples were: FM06 at 4,475 ± 28 N, FM08 at 5,864 ± 149 N, and FM10 at 7,024 ± 67 N. At 0.6 mm wall thickness, the specimens within the specified limits are: FM18 at 5,699 ± 113 N and FM20 at 6,068 ± 157 N. The 0.8 mm wall thickness specimens FM30 at 5,504 ± 49 N, and FM32 at 6,338 ± 122 N are within the specified limits. Table 4 provides a summary of these results.

**FIGURE 9:**
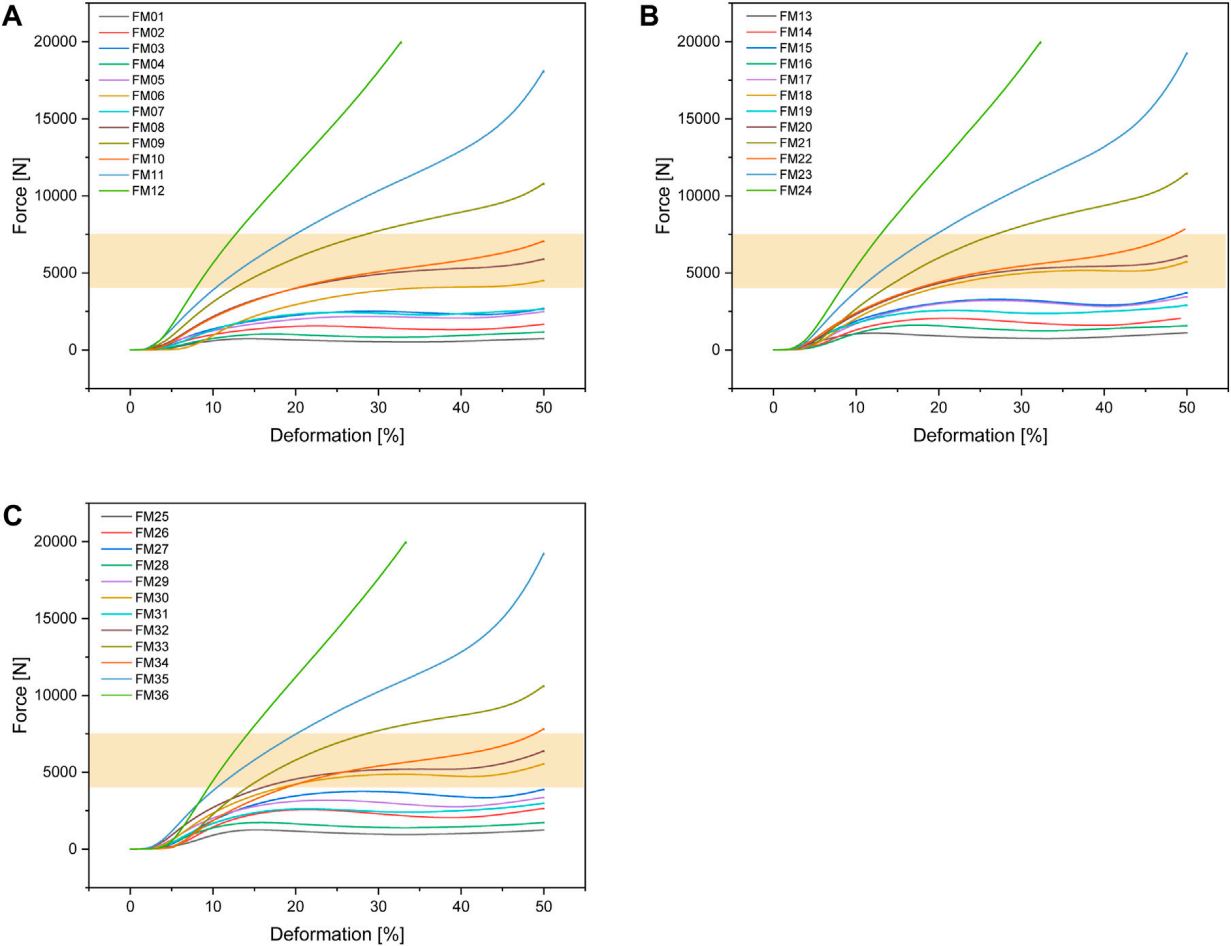
Force-deformation curves for the additively manufactured Gyroid structures out of FlexMed with **(A)** 0.4 mm; **(B)** 0.6 mm and **(C)** 0.8 mm outer wall thickness; orange area: target range for our disks between 4,000 and 7500 N, Approximately 35,000 measurement points for three samples each were averaged for the force/displacement curves.

Figure 9 shows the force-deformation curves for the FlexMed disks. As with FlexHard, we have again divided the curves according to the wall thickness of the outer wall (0.4; 0.6 and 0.8 mm) to provide a better overview.

With one exception (FM10), disks with Gyroid volumes of 6.8 and 10 mm³ and Gyroid wall thicknesses of 0.75 and 1.0 mm were within the target range regardless of disk wall thickness or filament used (FlexMed or FlexHard). Table 5 summarizes the results for those disks that were within the target range in the mechanical tests.

**TABLE 5:** Summary of disk dimensions that were within the target range (4,000–7,500 N) in mechanical tests.

FlexHard								
Outer wall 0.4 mm			Outer wall 0.6 mm			Outer wall 0.8 mm		
Sample	Gyroid volume mm³	Gyroid wall mm	Sample	Gyroid volume mm³	Gyroid wall mm	Sample	Gyroid volume mm³	Gyroid wall mm
FH06	8	1.0	FH15	10	1.0	FH27	10	1.0
FH08	6	0.75	FH17	8	0.75	FH30	8	1.0
			FH18	8	1.0	FH32	6	0.75
			FH20	6	0.75			
FlexMed								
FM06	8	1.0	FM18	8	1.0	FM27	10	1.0
FM08	6	0.75	FM20	6	0.75	FM30	8	1.0
FM10	4	0.5				FM32	6	0.75

### 3.3 Deformation behavior

**FIGURE 10:**
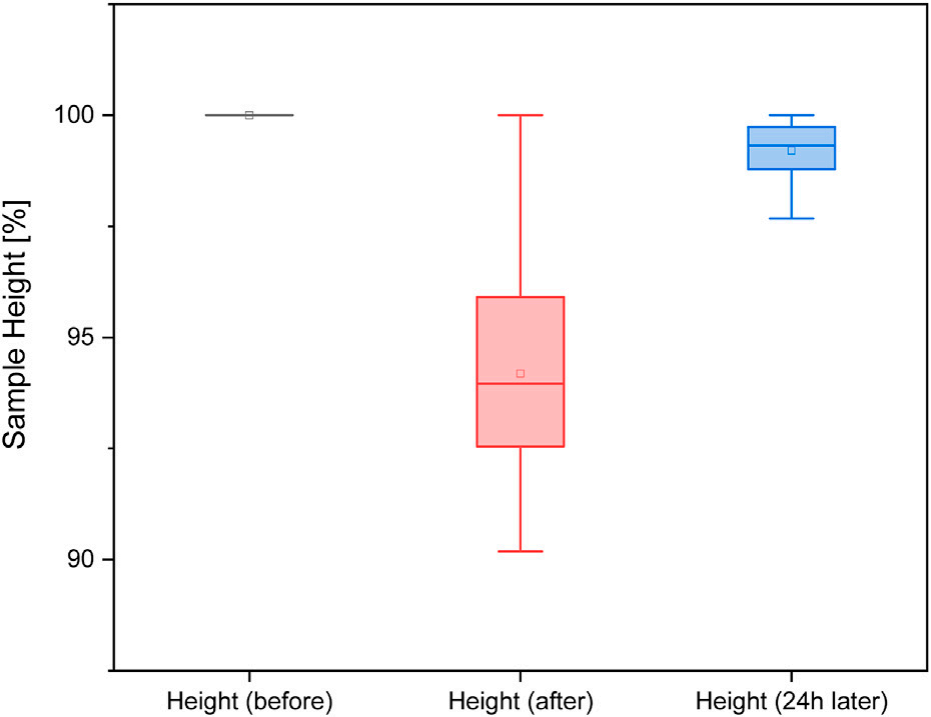
Sample height before, immediately after, and 24 h after mechanical test.

The sample heights directly after the mechanical measurement are slightly reduced by 5% compared to the initial heights. The sample height after 24 h is back to the initial value. With a significance level of *p* < 0.05, no significant difference was found between the two filaments (FlexMed and FlexHard). Therefore, the following Figure 10 was created from the height measurements of both filaments.

## 4 Discussion

The use of intervertebral disk prostheses has not yet found the acceptance on the current market that fusion surgery already has. This can also be confirmed by the number of operations performed in recent years. However, it should be borne in mind that patients who are implanted with a disk prosthesis are on average younger than patients who undergo spinal fusion. Another important aspect is that spinal fusion is associated with a significant restriction in the range of motion of the spine and the individual vertebral joints.

The large number of different disk prostheses in the cervical and lumbar region shows a trend towards articulating prostheses, which usually consist of one or two joints and use materials such as CoCrMo, Ti6Al4V or UHMWPE (Staudt et al., 2018; Sandhu et al., 2020). Only a few implants stand out from this group, for example, the Cadisk-C, which is a disk prosthesis in a monoblock design, similar to the disk in this paper. One advantage of a monoblock prosthesis is that the risk of wear particles or the release of metal ions is almost completely avoided due to the lack of articulation of two joint partners (Abi-Hanna et al., 2018; Sandhu et al., 2020). A monoblock constrained design may transfer shear stress to the interface between the vertebrae and implant, potentially causing implant migration and loosening (Staudt et al., 2018). Additionally, there is a risk of applying damaging tensile loads to the core during extension movements (Yue et al., 2009).

In the field of intervertebral disk prosthetics, there are already several prototypes and ideas for manufacturing prostheses using additive manufacturing with 3D printing. The IBAD prosthesis is one example. In theory, 3D printing of cell tissue can be used to print a living intervertebral disk that behaves, nourishes and renews itself in a similar way to the native disk (Li et al., 2010). The pilot project by Spetzger et al. (2016) is another possibility for using 3D printing in prosthesis production. However, the study refers to this prosthesis as a cage, as it has neither a joint nor cushioning properties. This model is a way of restoring the distance between the vertebrae rather than improving mobility. In their study, Domanski et al. (2015) also developed a disk prosthesis that was produced by additive manufacturing. They used both the SLM and FDM processes.

All of these prostheses produced using 3D printing have one thing in common: the use of CT or MRI scans of the patient to produce a customized disk prosthesis. A major problem in the current market is that prostheses are usually only available in a few different sizes. Patients who need a size outside the available range or have special requirements often have no way of obtaining a suitable disk prosthesis (de Beer and Scheffer, 2012). Another important aspect is that the wrong size of disk prosthesis is often associated with possible complications. The vertebral bodies should be completely covered by the disk prosthesis in order to prevent or reduce the risk of complications such as migration and heterotopic ossification. By using 3D scans of the patient, the disk prosthesis can be customized to each patient’s vertebral body shape. The use of 3D printing is a helpful method to quickly print models and make individual adjustments (Sandhu et al., 2020).

The first series of tests with FlexHard filaments showed that varying the size and wall thickness of the Gyroid produced different results in terms of structural integrity. Both the Gyroid volume and the wall thickness of the Gyroid played a critical role. It was clearly shown that the Gyroids that were too dense (small Gyroid volume of 4 mm³) were mostly well above the set limit of 7500 N. The wall thickness of the outer wall was much less important. Nevertheless, it is important to balance the interaction of Gyroid volume, Gyroid wall thickness and outer wall thickness for the specific application (cervical vs lumbar spine). The maximum load-bearing capacity of some specimens was higher than the theoretically calculated maximum loads of the intervertebral discs, indicating sufficient stability of the printed models (Rohlmann et al., 2001; Wang and Campbell, 2009; Abudouaini et al., 2023). The maximum strength of the vertebral bodies was defined as the upper limit of the examination. If the intervertebral discs exceed this value, this means that the intervertebral discs are stiffer than the surrounding bone of the vertebral bodies. This would be comparable to the stiffening of the vertebral bodies, which should actually be avoided with the additively manufactured Gyroid approach.

Measuring the height of the samples before and after the functional measurement shows that the 3D-printed parts largely return to their original shape even after compression. Measuring the flexible filament with calipers can lead to deviations, as some of the samples can be severely deformed by hand.

### 4.1 Limitations

One limitation to consider here is the low strain rate of the unconfined compression test of 5 mm/min, which is suitable for recording mechanical properties (DIN EN ISO 527). However, it is less suitable for simulating sudden loads such as those caused by a fall. In addition, some materials, including some polymers, exhibit strain-dependent mechanical behavior. However, it should also be noted that the present study was a feasibility study to determine the suitability of flex filaments for 3D printing Gyroid structures and their reproducible fabrication for biomechanical investigations. Dynamic fatigue tests or bending tests have not yet been performed, nor has the effect of wet conditions on mechanical properties (Xu et al., 2021) been determined - this will be investigated in a follow-up study with the new disc design.

## 5 Conclusions and outlook

The current state of the art shows that there is a wide variety of different disk prostheses. However, it has not yet been sufficiently researched whether a disk prosthesis should be preferred to spinal fusion. Another major problem is the medical complications that can be caused by incorrect implant selection or surgical methods. The aim of this work was to develop a disk prosthesis using 3D printing and Gyroid structures. The experimental results show that 3D printing is a promising method for producing individualized disk prostheses. Despite some technical challenges, 3D-printed prostheses offer a unique opportunity to improve spinal surgery. They allow customization to the individual needs of each patient. The wide range of possible applications is particularly noteworthy. The ability to create models based on CT or MRI scans enables precise replication of the natural disk structure. This allows an improved fit and function of the prostheses to be achieved and undesirable complications to be avoided.

**FIGURE 11:**
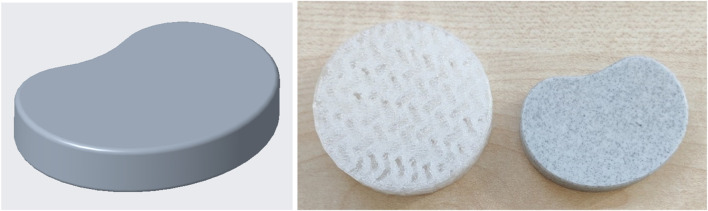
Possible new model for additive manufacturing from FlexMed and FlexSemiSoft for further static and dynamic mechanical investigations.

The flexible filaments used in this work still have room for improvement in terms of printing quality (especially the FlexSemiSoft, which is not listed here and was very difficult to print) and in terms of modifying the mechanical properties for modeling the disk prostheses. However, it has been shown that the flexible filaments used in this work have good mechanical properties and also exceed the specified limits. Another important feature is the model used in this work. It only remotely resembles a human intervertebral disk. Finally, another model was created and printed which resembles the human intervertebral disk much more closely than the one used previously (Figure 11). However, this new model still needs to be validated in a further study. In addition, fatigue strength tests are planned in a further project to ensure that the TPU/Flex filaments are also suitable for long-term use as intervertebral disc replacements. Further work could use CT and MRI scans to further increase the accuracy of the shape of the disk prosthesis.

## Data Availability

The raw data supporting the conclusions of this article will be made available by the authors, without undue reservation.
